# Comparison of Metabolite Concentrations in the Left Dorsolateral Prefrontal Cortex, the Left Frontal White Matter, and the Left Hippocampus in Patients in Stable Schizophrenia Treated with Antipsychotics with or without Antidepressants. ^1^H-NMR Spectroscopy Study

**DOI:** 10.3390/ijms161024387

**Published:** 2015-10-15

**Authors:** Dominik Strzelecki, Piotr Grzelak, Michał Podgórski, Olga Kałużyńska, Ludomir Stefańczyk, Magdalena Kotlicka-Antczak, Agnieszka Gmitrowicz

**Affiliations:** 1 Department of Affective and Psychotic Disorders, Medical University of Łódź, Central Clinical Hospital, Łódź 92-213, Poland; E-Mails: okaluzynska@gmail.com (O.K.); magdalena.kotlicka-antczak@umed.lodz.pl (M.K.-A.); 2 Department of Radiology-Diagnostic Imaging, Medical University of Łódź, Barlicki University Hospital No. 1, Łódź 90-153, Poland; E-Mails: piotr.grzelak@umed.lodz.pl (P.G.); chilam@tlen.pl (M.P.); ludomir.stefanczyk@umed.lodz.pl (L.S.); 3 Department of Adolescent Psychiatry, Medical University of Łódź, Central Clinical Hospital, Łódź 92-213, Poland; E-Mail: agnieszka.gmitrowicz@umed.lodz.pl

**Keywords:** schizophrenia, SSRI, SNRI, dorso-lateral prefrontal cortex, frontal white matter, hippocampus, ^1^H-NMR spectroscopy

## Abstract

Managing affective, negative, and cognitive symptoms remains the most difficult therapeutic problem in stable phase of schizophrenia. Efforts include administration of antidepressants. Drugs effects on brain metabolic parameters can be evaluated by means of proton nuclear magnetic resonance (^1^H-NMR) spectroscopy. We compared spectroscopic parameters in the left prefrontal cortex (DLPFC), the left frontal white matter (WM) and the left hippocampus and assessed the relationship between treatment and the spectroscopic parameters in both groups. We recruited 25 patients diagnosed with schizophrenia (DSM-IV-TR), with dominant negative symptoms and in stable clinical condition, who were treated with antipsychotic and antidepressive medication for minimum of three months. A group of 25 patients with schizophrenia, who were taking antipsychotic drugs but not antidepressants, was matched. We compared metabolic parameters (*N*-acetylaspartate (NAA), myo-inositol (mI), glutamatergic parameters (Glx), choline (Cho), and creatine (Cr)) between the two groups. All patients were also assessed with the Positive and Negative Syndrome Scale (PANSS) and the Calgary Depression Scale for Schizophrenia (CDSS). In patients receiving antidepressants we observed significantly higher NAA/Cr and NAA/Cho ratios within the DLPFC, as well as significantly higher mI/Cr within the frontal WM. Moreover, we noted significantly lower values of parameters associated with the glutamatergic transmission—Glx/Cr and Glx/Cho in the hippocampus. Doses of antipsychotic drugs in the group treated with antidepressants were also significantly lower in the patients showing similar severity of psychopathology.

## 1. Introduction

Schizophrenia is one of the most severe mental disorders, which affects around 1% of the population [1]. In periods of acute psychosis, therapeutic strategies focus on managing delusions, hallucinations, and formal thought and behavior disorders. In a stable state, the main therapeutic challenge is reduction of severity of negative (autistic behavior, flat affect, reduced social activities), affective (depressed mood, anxiety), and cognitive symptoms (attention deficits, impairment of working memory, and other executive functions). Dorsolateral prefrontal cortex (DLPFC) and hippocampus are specific brain regions essential for cognition and emotions [2,3,4,5]. Dysfunction of these regions is associated with presence of negative and cognitive symptoms. White matter (WM) within the frontal lobe consists of axons of cells that make up for the cortex, neurites projecting to the prefrontal cortex, and fibers connecting the DLPFC with the hippocampus [6].

Therapy with antidepressants (AD) is one of the most widely used methods for augmentation of antipsychotic treatment (AP). It aims to alleviate affective, negative, and to a lesser extent cognitive symptoms [7]. One meta-analysis demonstrated that significant efficacy of AD is observed particularly in patients with chronic phase of the disease [8], whereas conclusions of other studies are ambiguous [9,10]. Although it was believed that AD treatment may increase the risk of psychotic exacerbation, new data confirms that AD do not cause such complications [11].

Proton magnetic resonance spectroscopy enables *in vivo* assessment of brain metabolic parameters, including changes associated with drug administration. However, ^1^H-NMR studies in schizophrenia did not allow drawing definite conclusions about changes in metabolic parameters depending on the examined area of the brain, particular symptoms, and phase of the disease or treatment strategy. Most studies in schizophrenia showed a decrease in the concentrations of *N*-acetylaspartate (NAA) in gray matter, which is a ubiquitous brain metabolite, considered a reliable marker of viability and integrity of the brain tissue [12]. According to Brugger, Steen, and Kraguljac NAA concentration in the frontal cortex was similar in patients with the first episode of schizophrenia and individuals in a chronic phase of the disease [13,14,15]. Moreover it also did not depend on the duration of untreated psychosis (DUP) [16]. Concentrations of NAA and glutamatergic parameters may correlate with severity of the negative and cognitive symptoms [17,18,19,20,21,22,23,24]. There was a negative correlation between symptom severity and NAA concentrations in the prefrontal cortex, thalamus, and in the anterior cingulate [25,26,27,28]. Most studies, however, failed to demonstrate the relationship between concentrations of metabolites and exacerbation of clinical symptoms [29,30,31,32,33,34,35] or differences in NAA and Glu levels between patients with schizophrenia and healthy controls [36].

Findings in unmedicated patients with schizophrenia are also ambiguous. Kraguljac *et al.* found increased hippocampal Glx/Cr in treatment-naive patients but no alterations in NAA/Cr [37]. In study of Kegeles *et al.* elevated Glx levels in the medial prefrontal cortex were found [21]. Similar findings in dorsal caudate are also available [38]. Wood *et al.* noted no differences in NAA or Glx levels among similar patients when compared with healthy controls [39], while Tibbo *et al.* found no differences in glutamate levels between first-episode patients and a control group in the prefrontal region [40].

As far as the effect of medications is concerned, it was proved that AP affect concentrations of brain metabolites [33,41,42,43]. However, due to increasing number of patients with schizophrenia receiving AD (from 15% of patients in the 1990s to 40% in the last decade [44]) there is a need for evaluation of their influence. Effects of AD were already demonstrated in patients with major depressive disorder (MDD), who presented increased NAA/Cr ratios values in the left medial prefrontal cortex after successful treatment with selective serotonin transporter inhibitors (SSRIs) or serotonin and norepinephrine inhibitors (SNRI) compared to the pre-treatment values [45]. Moreover, in patients with major depression Block *et al.* described an association between treatment response to SSRI and tricyclic antidepressants and increase in NAA and Cho concentrations in the hippocampus, irrespective of the disease severity. Furthermore, it was revealed that low baseline NAA and Cho levels were predictors of the positive effects of the treatment [46].

We aim to compare ^1^H-NMR spectroscopic parameters (NAA, Glx, mI, Cho and Cr) in these regions of the dominant hemisphere that are crucial for pathogenesis of schizophrenia: the left DLPFC, WM of the left frontal lobe and in the left hippocampus between two groups of right-handed patients in the chronic phase of the illness with and without AD therapy. We hypothesize that there are differences in brain metabolic parameters between the two groups—with and without AD.

## 2. Results

There were no significant differences in patients’ clinical status and the results of PANSS (total score and subscales) and CDSS scales between both groups (Table 1 and Table 2). The only significant difference among the main analyzed parameters was the lower mean dose of AP administered in a group treated with AP+AD in comparison to patients receiving only AP. Antidepressive treatment is described in Table 3.

**Table 1:** Characteristics of groups.

Features		AP+AD Group	AP Group	*p* Value
		(*n* = 25)	(*n* = 25)	
Gender	Female	10	Female	0.5449
	Male	15	Male	
Age (years)		35.00	39.79	0.151
Mean number of hospitalizations		4	5	0.24
Mean duration of the illness (years)		12.1	13.7	0.1495
Mean timespan of education per patient (years)		14.7	13.8	0.5449
Antipsychotic treatment (DDD)		1.57	2.22	0.0195
Current smokers		11	9	0.5635

**Table 2:** Results of Positive and Negative Syndrome Scale (PANSS) and Calgary scales in each group.

Scales	AP+AD Group		AP Group		*p* Value
	Mean	SD	Mean	SD	
PANSS P	9.6	3.0	10.7	3.0	0.091635
PANSS N	25.0	4.6	26.4	5.3	0.462515
PANSS G	35.1	7.8	34.8	7.8	0.970098
PANSS Total	69.6	13.1	71.9	13.2	0.333484
Calgary	3.4	3.0	3.8	2.8	0.553671

PANSS P, N, G, Total: the Positive and Negative Syndrome Scale, Positive, Negative and General Psychopathology subscales and Total score; Calgary: Calgary Depression Scale in Schizophrenia score.

**Table 3:** Description of antidepressive therapy.

Type of Medication	Number of Patients	Mean Dose (mg)	Range (mg)
Sertraline	11	84.1	25–200
Citalopram	7	25.7	10–60
Fluvoxamine	2	75	50–100
Venlafaxine	2	225	225
Fluoxetine	2	15	10–20
Clomipramine	1	150	150
Escitalopram	1	10	10

The mean DDD of antidepressant was 1.42 (SD 0.87). One patient with severe negative symptoms received clomipramine with fluoxetine (20 mg).

Comparisons of spectroscopic data between both groups are presented in Table 4 (left DLPFC), Table 5 (left frontal WM), and Table 6 (left hippocampus).

**Table 4:** Comparison of substance concentration ratios in left DLPFC.

Parameter	AP+AD Group		AP Group		*p* Value
	Mean	SD	Mean	SD	
NAA/Cr	1.91	0.31	1.55	0.66	0.03651
Cho/Cr	0.68	0.24	0.76	0.35	ns
mI/Cr	0.26	0.11	0.28	0.13	ns
Glx/Cr	1.46	0.52	0.83	0.40	ns
NAA/Cho	2.19	0.61	1.48	0.72	0.01114
mI/Cho	0.38	0.15	0.48	0.57	ns
Glx/Cho	1.12	0.11	0.80	0.47	ns

NAA, *N*-acetylaspartate; Cr, creatine; Cho, choline; mI, myo-inositol; Glx, glutamate, glutamine and GABA; ns, not statistically significant.

In group AP+AD both NAA/Cr and NAA/Cho ratios (by 23.2% and 48%, respectively) were significantly higher (*p* < 0.05).

**Table 5:** Comparison of substance concentration ratios in left frontal WM.

Parameter	AP+AD Group		AP Group		*p* Value
	Mean	SD	Mean	SD	
NAA/Cr	1.79	2.16	1.62	0.96	ns
Cho/Cr	1.24	1.06	1.01	0.53	ns
mI/Cr	0.42	0.27	0.26	0.19	0.04291
Glx/Cr	0.83	0.26	0.69	0.27	ns
NAA/Cho	2.42	0.89	2.17	0.60	ns
mI/Cho	0.34	0.24	0.26	0.24	ns
Glx/Cho	0.73	0.25	0.81	0.24	ns

NAA, *N*-acetylaspartate; Cr, creatine; Cho, choline; mI, myo-inositol; Glx, glutamate, glutamine and GABA; ns, not statistically significant.

A typical glial parameter (mI/Cr) was significantly higher (by 61.5%) in the WM in AP+AD group.

**Table 6:** Comparison of substance concentration ratios in left hippocampus.

Parameter	AP+AD Group		AP Group		*p* Value
	Mean	SD	Mean	SD	
NAA/Cr	3.21	2.31	2.66	2.17	ns
Cho/Cr	0.97	0.72	1.45	0.88	ns
mI/Cr	0.74	0.72	0.62	0.85	ns
Glx/Cr	0.99	0.37	1.42	0.63	0.04232
NAA/Cho	3.48	4.10	1.70	0.94	ns
mI/Cho	2.46	5.48	0.44	0.68	ns
Glx/Cho	1.00	0.39	1.84	0.69	0.01703

NAA, *N*-acetylaspartate; Cr, creatine; Cho, choline; mI, myo-inositol; Glx, glutamate, glutamine and GABA; ns, not statistically significant.

In the left hippocampus both parameters associated with the glutamatergic transmission were significantly lower in the group treated with AP+AD than in patients receiving only AP (by 30.3% in Glx/Cr and by 45.6% in Glx/Cho ratio).

Age and smoking status may also influence metabolite concentrations [47,48,49]. Thus, both factors, together with group affiliation, were defined as independent variables in multiple stepwise regression analysis. In this analysis technique only independent variables that influence a dependent variable are included into the model. In Table 7 there are presented variables that were included in the model for each metabolite concentration ratio in particular brain regions. For brain metabolites not presented in the table the regression analysis did not include any variable into the model.

**Table 7:** Multiple stepwise regression analysis of the determinants of substance concentration ratios in left DLPFC, frontal WM and hippocampus.

Brain Region	Concentration Ratio	Predictor	β-Coefficient (±SD)	Corrected *R*^2^ of the Model	*p* Value
DLPFC	NAA/Cr	AD	0.3650 (0.2135)	0.05354	0.0967
	Cho/Cr	Age	0.0089 (0.0052)	0.05587	0.0950
		Smoking	−0.1189 (0.1050)		0.2661
	Glx/Cr	AD	0.6290 (0.3549)	0.26304	0.1365
	NAA/Cho	AD	0.7744 (0.3095)	0.1696	0.0177 *
		Age	−0.0204 (0.0142)		0.1595
	Glx/Cho	Age	0.0229 (0.0170)	0.1207	0.2347
White Matter	NAA/Cr	AD	0.5053 (0.1645)	0.2966	0.0063 *
	mI/Cr	AD	0.1506 (0.0796)	0.0705	0.0674
	Glx/Cr	AD	0.1282 (0.1235)	0.0396	0.3129
		Age	0.0056 (0.0051)		0.2795
		Smoking	0.1756 (0.1311)		0.1972
	NAA/Cho	AD	0.33926 (0.2874)	0.0479	0.2463
		smoking	−0.43080 (0.2765)		0.1288
	mI/Cho	AD	0.1002 (0.0795)	0.02825	0.2156
		Smoking	−0.0949 (0.0758)		0.2192
Hippocampus	Cho/Cr	AD	−0.6074 (0.3218)	0.0960	0.0718
		Smoking	−0.3529 (0.3169)		0.2769
	Glx/Cr	Age	−0.0219 (0.0103)	0.1666	0.0478 *
		Smoking	0.3640 (0.2354)		0.1394
	NAA/Cho	AD	8.7339 (4.8314)	0.0980	0.0822
		Age	0.2934 (0.2223)		0.1985
	mI/Cho	AD	2.492 (1.3188)	0.1604	0.0709
		Age	0.1294 (0.0633)		0.0519
	Glx/Cho	AD	0.8307 (0.2958)	0.3646	0.0170 *

NAA, *N*-acetylaspartate; Cr, creatine; Cho, choline; mI, myo-inositol; Glx, glutamate, glutamine and GABA; AD, antidepressive treatment; Smoking, smoking statutus; SD, standard deviation; *, statistically significant.

## 3. Discussion

This is the first study comparing spectroscopic parameters in two groups of patients with schizophrenia differing by the addition of AD to AP treatment. In discussion, we will focus on relevant results depending on the region where these changes were observed.

### 3.1. NAA and DLPFC

*N*-Acetylaspartate is one of the most common amino acids in the human brain. The role of the NAA in the brain is uncertain, but it is probably engaged in the metabolism of other amino acids (glutamate) and fluid balance [12]. It is specifically synthesized and present in high concentrations in neuronal mitochondria, but not in glia. Moreover, its level is closely associated with neuronal glucose metabolism [50]. Thus, NAA is considered as a marker of neuronal viability and integrity [12]. A higher NAA/Cr and NAA/Cho ratio observed in DLPFC in the AP+AD patients suggests that overall neuronal activity in this area was increased when compared to the AP group. As we previously mentioned, the role of the decreased activity of DLPFC in development of negative and cognitive symptoms is well established [51,52]. We suspect possible positive influence of AD on DLPFC metabolism and clinical improvement in context of our findings, even if efficacy of AD in treating negative and cognitive symptoms is still uncertain [11,53]. Methodological characteristics of our study (one time point assessment) does not allow provision of an unambiguous answer, whether AD improved mentioned symptoms. An increase of NAA concentration can be an effect of the antipsychotic drugs [33]. A definitive conclusion should be formulated carefully in this case, but the absence of other statistically significant variables indicates that the use of lower doses of AP may be associated with the use of AD.

In regression analysis we confirmed that administration of AD has the biggest influence on changes in NAA/Cr and NAA/Cho ratios. Although, age was included to the model for NAA/Cho it had small β-coefficient (−0.0204).

### 3.2. Glx and Hippocampus

It is postulated that in schizophrenia, glutamatergic system hyperactivity results from an impaired control of GABAergic interneurons and disturbed interactions with dopaminergic system [54].

A decrease in Glx/Cho and Glx/Cr ratios in the experimental group may indicate a positive effect of AD in the management of schizophrenia, because an increase of glutamatergic transmission parameters in hippocampal formation was previously observed in schizophrenia [18,37,55,56]. It was speculated that a psychotic process originates from the impaired glutamatergic transmission within the dentate gyrus in the hippocampus complex, which in consequence, leads to the hyperglutamatergic state within the CA1, CA3 fields and the subiculum of the hippocampus [57,58]. Functional changes in CA3 neurons may cause increased neuronal excitation, inadequate plasticity changes, such as disturbances of a long-term potentiation process (LTP) in aforementioned subfield [57,59].

Having the source in the dysfunctional inhibitory processes, which in schizophrenia results from hypofunction of NMDA receptors on GABAergic interneurons [60], an increased glutamatergic stimulation in the hippocampus is considered one of the key causes of attention deficit and cognitive dysfunction [57]. Cognitive function decline (assessed with the Wisconsin Card Sorting Test) was correlated with increased glutamate concentrations in the hippocampus in patients with schizophrenia, but not in healthy controls [60].

Insufficient control of the excitatory glutamatergic system may be also responsible for the development of hallucinations, delusions, and formal thought disorders typical for the acute psychotic states [54]. These inhibitory control dysfunctions may be expressed in gamma rhythms (25–100 Hz) changes [61,62], coherent neuronal oscillation disturbances (rate below 0.1 Hz) [63] which both might resemble increased information redundancy.

Our results indicate that in AP+AD group pathological hyperactivity within the hippocampus is reduced. It is the first evidence that unfavourable increase of the glutamate concentration within the hippocampus in schizophrenia is slighter in AP+AD group than in AP group.

According to the regression analysis, AD administration had significant effect only on Glx/Cho ratio, but not on Glx/Cr, which was more dependent on age and smoking status. On the other hand, the model for Glx/Cr explained only 16.7% of its variation and 36.5% for Glx/Cho. Thus, AD seems to have significant influence on hippocampus metabolism.

### 3.3. mI and WM

Myo-inositol—a precursor in the phosphatidylinositol signalling system is also a widely accepted glial marker in spectroscopic studies [64]. Generally, in neurodegenerative processes, increased mI levels have been found to co-occur with reduced NAA [65]. In these cases increased mI concentration is probably associated with neuroinflammation, neuronal damage, and glial proliferation [46]. Interestingly, current and previous depressive episodes in patients with schizophrenia or schizoaffective disorder may be related to higher levels of mI comparing to patient with schizophrenia without depressive episode [66]. In this study, the duration of the disease and its severity were comparable between both groups. Thus, it can be assumed that there were no significant differences in the intensity of glial proliferation due to the illness and that the observed differences were probably related to the treatment.

We suspect that increased mI/Cr ratio in the group treated with antidepressants may indicate higher activity of glial cells, associated with overall improvement of the brain metabolism, observed in other brain area or mI tendency to increase in patients with affective features having AD treatment as in study of Chiapelli *et al.* [66]. Although AD were included in the model for mI/Cr ratio this variable was not significant (0.0674). Surprisingly, AD were significant determinants of NAA/Cr ratio explaining almost 30% of its variance. This influence might be missed in initial comparison due to masking effect of age and/or smoking status.

### 3.4. Doses of AP

Difference in the mean doses of the antipsychotic drug, significantly lower in AP+AD patients, requires a commentary. Our data is insufficient to draw convincing conclusions, but this may indicate that to achieve similar parameters of the mental state using AD and AP, reduced doses of AP are needed.

## 4. Materials and Methods

### 4.1. Materials

Subjects with schizophrenia, aged 18–60 years who were physically, neurologically and endocrinologically healthy and had normal laboratory values (routine blood tests, biochemical tests including TSH, lipid profile, liver and kidney parameters, and ECG) were eligible to enter the study. Patients in acute psychosis, on clozapine treatment or declaring suicidal tendencies, were excluded from the study. This research is a part of Polish Sarcosine Study in Schizophrenia (PULSAR), for further details please see acknowledgments.

### 4.2. Methods

Fifty right-handed patients diagnosed by two experienced psychiatrists with schizophrenia with dominant negative symptoms according to DSM-IV-TR criteria who were in a stable clinical condition had ^1^H-NMR spectroscopy according to the protocol described below. 25 patients were treated with antipsychotics and antidepressants (AP+AD group), remaining subjects (AP group) received only antipsychotics (characteristics of each group is presented in Table 1). All patients were given stable doses of drugs for a minimum three months before a visit. Doses of AP and AD were calculated for defined daily dose (DDD), developed by the World Health Organization. Severity of schizophrenia symptoms was assessed with the Positive and Negative Syndrome Scale (PANSS) [67] and the Calgary Depression Scale in Schizophrenia (CDSS) [68].

### 4.3. Spectroscopy

Imaging was performed using 1.5 Tesla (T) MR scanner (Siemens Avanto 1.5, Munich, Germany) equipped with a standard head coil. NMR acquisition:

(1) FLAIR sequences in axial plane with following parameters: Repetition Time (TR), 9000 ms; Echo Time (TE), 105 ms; inversion time (TI), 2500 ms; flip angle, 150°; voxel size 1.4 × 1.3 × 3 mm.
(2) T2-weighted sequences were obtained in coronal plane with following parameters: TR = 5000 ms; TE = 100 ms; flip angle, 50°; voxel size 0.6 × 0.6 × 5.0 mm.
(3) T1 weighted sequences in transverse plane with following parameters: TR = 400 ms; TE = 7.8 ms; flip angle, 90°; voxel size 0.9 × 0.9 × 0.5 mm.

**Figure 1:**
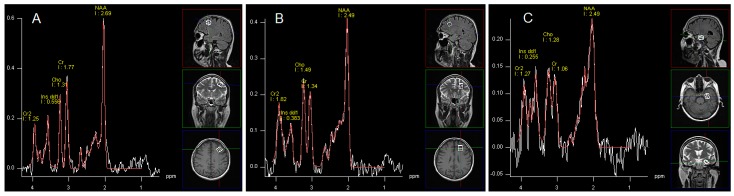
Images showing voxel location in the left DLPFC area (**A**), left frontal WM (**B**), left (**C**) and an examples before (white line) and after (red line) fitting. Peak areas for *N*-acetylaspartate (NAA); creatine (Cr and Cr2); choline (Cho); and myo-inositol (mI dd1) are labelled.

^1^H-MRS data were acquired by single voxel spectroscopy (SVS) using a point resolved spin echo (PRESS) sequence 128 averages; TR, 3000 ms; TE = 30 ms; voxel size was 15 × 15 × 15 mm for the left DLPFC, the left frontal WM and 10 × 15 × 20 mm for the left hippocampus. Regions of interest were placed in: the left DLPFC, left frontal WM and left hippocampus by a neuroradiologist (Figure 1). Automated procedures were used to optimize radiofrequency pulse power, field homogeneity, and water suppression, as well as to convert the lines into a Gaussian shape. Post-processing of spectroscopy data was performed by means of Avanto Syngo MR Software (Siemens, Munich, Germany), Level B15. It included: k-space Fourier transformation and a spatial 50 Hz Hanning filter; subtraction of the residual water signal; time domain 1 Hz exponential apodization; zero filling to 2048 points; Fourier transformation of the time domain signals; frequency shift correction, phase correction, and baseline correction. In postprocessing software also provided information about fitting error (deviation between theoretical and measured spectrum determined using the last squares method). We assumed that only spectra with fitting error less than 0.5 would be considered in the analysis. If the error was higher, the sequence was repeated. Hence, in all patients we received satisfactory values (mean fitting error 0.27 SD 0.12). The following metabolites were assessed: *N*-acetylaspartate (NAA); glutamine, glutamate and partially GABA (Glx); myo-inositol (mI); choline-containing compounds (Cho); creatine plus phosphocreatine (Cr). No absolute concentrations of metabolites were determined, but ratios to Cr and Cho.

### 4.4. Statistical Analysis

Continuous variables are expressed as the mean ± standard deviation (SD). The Shapiro-Wilk test was used to determine the normality of the data distribution. The χ^2^ test with Yates or Fisher corrections was applied to compare differences in qualitative data between groups. Continuous variables were analyzed by means of the Mann-Whitney test. Due to the fact that concentrations of brain metabolites may be affected by other factors than AD administration, multiple stepwise regression analysis was performed. Independent variables included: group affiliation (experimental *vs.* control), age, smoking status, and DDD of antipsychotics. Statistical analysis was performed using Statistica for Windows (version 12.0, StatSoft, Tulsa, OK, USA). A *p*-value of ≤0.05 was considered significant.

## 5. Limitations of the Study

The main limitations of the study are:

(1) Application of a 1.5 T magnetic field, rather than a 3 T (or stronger) field. A stronger magnetic field would allow overlapping peaks of glutamine, glutamate, and GABA to be distinguished.
(2) Calculation of ratios instead of using exact concentrations. However, Cr or Cho levels remain stable in the course of schizophrenia treatment [69,70], thus our conclusions seem to be reliable.
(3) In our study, we assessed patients only in one point of time. Hence, we do not know what was the basics characteristic of groups before administration of AD in the AP+AD group. It is possible that these patients presented more severe negative symptoms and that difference in PANSS and Calgary scores would be significant. However, similar clinical presentation observed in both groups, with significantly lower doses of AP administrated in AP+AD group, indicates that AD are a promising therapeutic option that require further investigation.
(4) Finally, we compared a few metabolites ratios between groups. Thus due to I type error presented significant results could be obtained coincidentally. If we applied the Bonferroni correction for multiple testing the corrected p would be 0.05/21 = 0.002, thus all the results would be insignificant. On the other hand, these results are clinically significant and biologically relevant, hence further evaluation with more powerful tests, and within greater population, are required.
(5) In addition to age and smoking status included in the multiple testing, AP treatment also could have affected the tested parameters. AP treatment was not homogenous in our groups and differed significantly in terms of DDD, which must be mentioned in the section describing limitations of our study.

## 6. Conclusions

We observed a trend toward increased values of parameters of neuronal viability in DLPFC and overall brain metabolism and reversed glutamatergic overstimulation in hippocampus in patients with augmentation of antipsychotic treatment with antidepressants, when compared to AP group parameters. Moreover, in the AP+AD group reduced doses of AP were used, which in consequence may reduce the risk of side effects. Although we observed parameter differences between two study groups, we cannot make reliable conclusions about influence of AD on patients’ clinical status and this issue require further investigation. Further prospective studies are warranted to determine more precisely the effects of antidepressants on spectroscopic parameters.
